# Anthrax and Preventive Inoculation in Louisiana1Read before the Thirty-eighth Annual Meeting of the American Veterinary Medical Association.

**Published:** 1901-11

**Authors:** W. H. Dalrymple

**Affiliations:** Baton Rouge, La.


### ANTHRAX AND PREVENTIVE INOCULATION IN LOUISIANA.[1]

By W. H. Dalrymple, M.R.C.V.S.,
BATON ROUGE, LA.

(Concluded from page 618.)

Preventive inoculations were first made by Toussaint, but were apparently unsuccessful in obtaining the desired results. Pasteur, however, demonstrating that immunity was produced by weakened virulence on the part of the organism, obtained an attenuated virus by cultivating the bacterium at a temperature of 42° to 43° C. in the presence of oxygen. There are other processes of the preparation of the virus, but the lymph that I am most familiar with and

[1] Read before the Thirty-eighth Annual Meeting of the American Veterinary Medical Association.

the one which has been put into practice in almost all Continental European countries is that prepared by the Pasteur method. The dose of this virus comprises two inoculations. The first lymph, or first half of the dose, is that which has been cultivated, under the conditions above mentioned, for about twenty-four days; the second lymph for about twelve days. The first dose is, therefore, the more attenuated, and seems to be somewhat preparatory to the second, which is more powerful. An immunizing dose requires one-quarter of a cubic centimetre of each lymph given from ten to fourteen days apart.

The records of preventive inoculation by the use of the attenuated virus, especially in European countries, seem to be extremely gratifying. And I think I can confidently assert that excellent results have been obtained in Louisiana when all the conditions were favorable, such as good material, strict antiseptic care observed in its use, and the operation performed early in the season, so as to secure immunity before the advent of the hot months, during which the disease usually makes its appearance.

With the exception, perhaps, of a very few individuals planters who imported virus direct from France, preventive inoculation against anthrax was but little known or practised in Louisiana previous to 1896. In that year we had an extensive outbreak in the northern portion of the State, but in a section containing all the conditions favorable for the propagation of horse-flies to carry abroad the infection, and I certainly never before witnessed such a "fly plague." It was at this time that I suggested the use of the anthrax virus, and the doses used that season throughout the State amounted up to some thousands. Since then the use of the lymph has been fairly general, especially in the localities in which anthrax had been prevalent, as well as in those contiguous, until, during the past summer, the large number of about thirty thousand doses of the Pasteur lymph alone have been used.

It is difficult to obtain accurate data with regard to the positive results of preventive inoculation in Louisiana, the work of vaccinating having fallen, to quite a large extent, into the hands of those with a very limited knowledge, if any, of the importance of strict antisepsis. Some of the untoward results arising in consequence may here be cited. Extensive septic infection from dirty instruments, etc., and in some cases from infection of the virus through careless handling, such as frequently opening the vial before its contents were all used up; using wrong virus—I have it on good authority that black-leg vaccine has been used on several occasions for anthrax lymph, the reason for the error being, I presume, due to the fact that the black-leg vaccine is frequently labelled for symptomatic charbon or anthrax, and the operators being ignorant of the difference. Quite recently I have observed through our daily press that a number of cases of tetanus had followed vaccination for anthrax. It is difficult to account for this, except through the proper precautions being neglected during the operation; through after-infection of the inoculation punctures, or through virus infected with tetanus organisms. If the latter, it shows how completely the veterinarian is dependent upon the reliability of the manufacturer and vendor of these products. Want of due protection and care of inoculated animals before complete immunity is established in the midst of an epizoöty of anthrax has also brought about indifferent results, etc.

The best season to vaccinate in our climate is almost any time before the heated term, so as to permit of complete immunity before the climatic conditions appear that are favorable to the development of bacterial and insect life.

Personally, I have vaccinated only a few hundred head of stock, the most of the work in recent years being done by practising veterinarians, managers and owners of plantation and farm stock, a few physicians, and a number of "quack vaccinators." Although I wrote to several practitioners in the State for the results of their experience with preventive vaccine, I failed to get a response in the majority of instances.

I have a record from a physician who assisted in inoculating stock in the 1896 epizoöty in North Louisiana. During the height of the outbreak he vaccinated some two hundred and fifty head of horses and mules with the Pasteur lymph. After the first inoculation about 3 per cent. showed symptoms of the disease, but not more than 50 per cent. of the 3 per cent. died. After the administration of the second lymph there were no more deaths, with only a few animals exhibiting symptoms. At the same time, however, unvaccinated animals in the neighborhood were dying with great rapidity. During this same outbreak there were other somewhat similar records, but I have not the exact data. In the spring of 1897 I vaccinated over two hundred horses and mules in one locality without a single case, so far as I know, of œdema larger than a pigeon's egg at the point of inoculation. Vaccination has been carried on since in this vicinity, but I have not heard of a single death, although the disease was in the neighborhood this summer.

In 1899 the owner of five large sugar plantations, with an aggregate of 368 mules, furnished me with detailed statistical results of vaccination that summer, a summary of which is as follows: 39 cases and 14 deaths, or average number of animals taken sick, 10.6 ; average number died, 3.8 ; average number of deaths of sick animals, 35.9. During the 1899 outbreak, and while making some investigations into the history of anthrax in different portions of the State, the owner of a large plantation, in a charbonous district below the city of New Orleans, informed me that he had vaccinated about one hundred mules in the previous five years, and had lost only two animals during that time, one not inoculated, the other permitted to graze on a headland over which anthrax carcasses had previously been dragged. In the summer of 1899 the disease was epizoötic all around the neighborhood of this plantation, with flies excessively numerous, such being always the case during the years in which we have the most wide-spread outbreaks.

In my own parish of East Baton Rouge we had that summer a few cases transmitted from a neighboring parish in which a large number of all kinds of animals were lost. Precautions were at once taken, through the local authorities of both parish and city, to cremate all victims known to have died of anthrax and to inoculate extensively. This put a check to the spread, but early in the spring of 1900 a bull which had been roaming over the locality where the disease had appeared the previous summer died near our experiment station (to which case I have already alluded), and was the means of causing the death of a cow, a horse, and a mule belonging to the station. Every precaution was taken to prevent further disaster, such as cremation, thorough disinfection, and the inoculation of some fifteen to twenty head of the remaining stock. And although the animals were turned back on the pasture where the first case occurred, about two weeks after immunity was established, there has not been a suspicious case on the place since, a period of about sixteen months.

The past summer the disease broke out in two wards of our parish. Strict attention was at once given to cremation or deep burial of the carcasses, as well as inoculation, and the disease was checked in each instance. One ward lost probably ten animals before proper measures were adopted, and the other ward lost one mule.

An extensive landowner and merchant of my acquaintance vaccinated this summer 127 head of mules after anthrax had broken out close to his property. I saw the animals about ten days after they had had the second lymph, and up to that time he had not lost a single one.

Dr. E. Pegram Flower, a graduate practising in the city of Baton Rouge, inoculated about 2400 animals, chiefly mules, the past summer in our State, and about 500 in the Mississippi delta, all being exposed to infection—*i. e.*, the disease was prevalent all around the vicinity. His losses in Louisiana amounted to not more than one-quarter of 1 per cent., while in Mississippi only 7 animals out of the 500 died after inoculation was commenced, notwithstanding the fact that over 100 died in the neighborhood previous to inoculation, the infection being of such a virulent character.

In the summer of 1899 this same gentleman inoculated some 1800 head of stock in our State, and with a loss of not over 1 per cent., which seems to me to be a very satisfactory showing in favor of the use of the preventive vaccine.

Perhaps the most convincing evidence of the beneficial effect of this method of prevention in Louisiana is the fact that those localities which suffered most from yearly, or at least periodic, epizoötics of anthrax, before vaccination became so generally adopted, experienced the past summer a wonderful degree of immunity from the disease, which, I think, we must attribute to the fact that the use of the lymph is now almost general in those sections and that greater attention is being directed to the more careful disposal of the dead animal, our people more fully appreciating its being the chief source from which this most deadly disease is spread.

I believe we are gradually solving the anthrax problem in the Pelican State, and the progress we have already made is, I think, considerable and fairly satisfactory when we take into account the amount of ignorance, superstition, and the erroneous and visionary ideas which prevailed up to ten or twelve years ago regarding the true nature of the disease and the most potent factors in causing its spread. What we have accomplished has in great measure, I think, been due to a persistent endeavor to educate our people, for we have no sanitary laws of much importance and no livestock sanitary board or commission vested with authority to properly execute those we have. This condition of affairs, however, we hope to see changed in the near future. I question very much if ten years ago a single dose of preventive vaccine was used, or an anthrax carcass destroyed as a sanitary precaution against the spread of the disease in our State. To-day there are probably 40,000 or 50,000 doses of vaccine used, and carcasses are being much more carefully looked after, which I feel indicates some progress at least.

With years of added infection in our anthrax localities, and with such favorable climatic conditions for bacterial and insect development as we possess, complete extermination of the infection cannot be looked for in the immediate future. So we must endeavor to live among it by rendering ourselves immune against it until such conditions arise by which we can stamp it out. Our measures must be preventive and strictly sanitary, the importance of both of which we have been trying to impress upon our authorities and people. First of all, preventive inoculation in the hands of competent individuals, the careful and proper disposal of all anthrax carcasses, and thorough disinfection.

The question of the destruction or extermination of the horse-fly in the swampy or moist sections of our State is an important one and should be taken up and thoroughly investigated by the entomologist, either State or national. But, so far as I am able to see, there are only two methods by which the problem might be solved: one is to thoroughly drain and reclaim such localities, which I expect will be accomplished some day; and the other, although rather more unlikely, is to turn on one of our now famous "oil-gushers" and destroy the flies in their watery haunts with mineral oil, as Porchinski succeeded in doing in the forest pools in Russia.

